# Genome-Wide Identification of the *Nramp* Gene Family in *Spirodela polyrhiza* and Expression Analysis under Cadmium Stress

**DOI:** 10.3390/ijms22126414

**Published:** 2021-06-15

**Authors:** Yan Chen, Xuyao Zhao, Gaojie Li, Sunjeet Kumar, Zuoliang Sun, Yixian Li, Wenjun Guo, Jingjing Yang, Hongwei Hou

**Affiliations:** 1 The State Key Laboratory of Freshwater Ecology and Biotechnology, The Key Laboratory of Aquatic Biodiversity and Conservation of Chinese Academy of Sciences, Institute of Hydrobiology, Chinese Academy of Sciences, Wuhan 430072, China; chenyan@ihb.ac.cn (Y.C.); zhaoxuyao@ihb.ac.cn (X.Z.); ligaojie@ihb.ac.cn (G.L.); kumar.sunjeet082@gmail.com (S.K.); zlsun@ihb.ac.cn (Z.S.); liyixian@ihb.ac.cn (Y.L.); guowenjun@ihb.ac.cn (W.G.); 2 University of Chinese Academy of Sciences, Beijing 100049, China; 3 College of Environment and Chemical Engineering, Pingdingshan University, Pingdingshan 467000, China

**Keywords:** *Spirodela polyrhiza*, *Nramp* family, promoter, Cd, Fe or Mn limiting, gene expression profiling

## Abstract

Natural resistance-associated macrophage proteins (Nramps) are specific metal transporters in plants with different functions among various species. The evolutionary and functional information of the *Nramp* gene family in *Spirodela polyrhiza* has not been previously reported in detail. To identify the *Nramp* genes in *S. polyrhiza*, we performed genome-wide identification, characterization, classification, and cis-elements analysis among 22 species with 138 amino acid sequences. We also conducted chromosomal localization and analyzed the synteny relationship, promoter, subcellular localization, and expression patterns in *S. polyrhiza*. β-Glucuronidase staining indicated that *SpNramp1* and *SpNramp3* mainly accumulated in the root and joint between mother and daughter frond. Moreover, *SpNramp1* was also widely displayed in the frond. *SpNramp2* was intensively distributed in the root and frond. Quantitative real-time PCR results proved that the *SpNramp* gene expression level was influenced by Cd stress, especially in response to Fe or Mn deficiency. The study provides detailed information on the *SpNramp* gene family and their distribution and expression, laying a beneficial foundation for functional research.

## 1. Introduction

Natural resistance-associated macrophage proteins (Nramps), which comprise a highly conserved gene family across all species, from bacteria to humans, and are identified as an integral membrane protein family, are critical proton/metal transporters in plants [1,2,3,4]. The genes of the *Nramp* transporter family play important roles in the transport of ions, such as Cd, Fe, Mn, and Zn [5,6,7,8,9,10]. In *Arabidopsis thaliana*, *AtNramp1* is located in the plasma membrane and is identified as an Fe, Mn, and Cd transporter [11,12], whereas *AtNramp2* is located in endomembrane and acts in the distribution of Mn between intracellular organelles [13,14]. *AtNramp3* and *AtNramp4* participate in the vacuolated export of Mn during seed germination, and function in photosynthesis and growth under Mn deficiency [15,16]. *AtNramp5* is involved in the transport of Cd and Fe [17]. *AtNramp6* is involved in the intracellular transport of Cd [18]. In rice, the *Nramp* genes transport Fe, Cd, As, Mn, and other ions. *OsNramp1* encodes a transport protein of Fe, As, and Cd, but not Mn [6,19,20], while *OsNramp3* transports Mn but not Fe or Cd [21]. Besides, *OsNramp4* is a transporter for Al that cannot transport divalent cations in yeast [22], whereas *OsNramp5* is an Fe, Mn, and Cd transporter [23,24]. *OsNramp6* is a plasma membrane-localized protein that acts as Fe and Mn transporters [25], whereas *OsNramp2* is induced by high Cd accumulation in the shoots [26]. The overexpression of some *Nramp* genes from *Thlaspi caerulescens* (*TcNramp3* and *TcNramp4*), *Crotalaria juncea* (*CjNramp1*), and *Thlaspi japonicum* (*TjNramp4*) enhance the accumulation of ions, such as Cd, Fe, and Ni [12,27,28]. By contrast, *MhNramp1* from *Malus hupehensis* leads to cell death by Cd uptake in tobacco and apple calli [29]. Ethylene-insensitive protein 2 (EIN2), a unique membrane-anchored protein, is similar to the eukaryotic Nramp family of metal ion transporters with a 12-membrane span, and it is important for stress response and ethylene-signaling pathways [2,30].

Reports on *Nramp* genes have shown their diverse functions in different species in metal ion transport. Over the past few years, people have focused more on metals in the aquatic environment [31,32], especially Cd. Although Cd is not an essential nutrient element, it could be absorbed directly by plants from the environment, transferred to human beings through the food chain, and may thus cause severe damage to human health, even at low concentrations [33,34,35].

In comparison with terrestrial plants, aquatic plants suffer from more damage under improper metal ion concentration when the whole plant is exposed to the environment [36,37,38,39,40]. Therefore, the absorption and transportation mechanism of toxic metal ions in aquatic plants should be studied. *S. polyrhiza*, a monocotyledonous free-floating named giant duckweed, is widely distributed throughout the world, and represents the largest duckweed (1.5 cm long) with the smallest genome size (158 Mb) among all duckweeds measured [41]. Moreover, the genome of *S. polyrhiza* has been estimated, and the annotation has been further refined, thus providing a convenient model for molecular research [41,42,43,44], including the molecular mechanism studies of metal ion resorption and excretion [45,46,47,48,49,50].

Up to now, many *Nramp* gene families have been characterized and analyzed, while the *Nramp* genes of evolutionary and functional information under heavy metal stress in *S. polyrhiza* are rarely reported. To reveal the function of *Nramp* genes response to Cd stress, we identified the *Nramp* genes in *S. polyrhiza*, constructed a phylogenetic tree, analyzed phylogenetic relationships, gene structures and features, cis-acting elements, and conserved motifs with 21 other species. We also investigated the structural characteristics and physicochemical properties among all selected species, and then determined the colinear relationships among nine species. To effectively understand the function of *S. polyrhiza*, we carried out promoter analysis by fusion protein expression of β-glucuronidase (GUS). Furthermore, we analyzed the expression patterns of *SpNramp* genes under several heavy metal stressors.

Promoter analysis results indicate that *SpNramp1*, *SpNramp2*, and *SpNramp3* highly accumulated under Cd stress. According to quantitative real-time PCR (qRT-PCR) results, the relative expression level was influenced by Cd stress and Cd stress with Fe^2+^ or Mn^2+^ deficiency in *S. polyrhiza*. To date, the relative study of *SpNramp* genes has been rarely reported. Our findings provide a foundation for future investigation on the role of *Nramp* genes in *S. polyrhiza*.

## 2. Results

### 2.1. Identification of Nramp Genes in S. polyrhiza

*Nramp* genes in 22 species were identified using BLAST-HMMER methods. We had divided the protein into the Nramp family with the conserved Nramp domain and several transmembrane regions, including the Nramp metal transporter family and EIN2 [2]. After the deletion of repetitive and short coverage sequences, 138 amino acid Nramp sequences were analyzed, as shown in Supplemental Materials Table S1. We named *Nramp* genes based on the order of gene location in chromosomes or scaffolds, except for those that have been named in the previous studies. Retrieved protein sequences included 1, 2, and 3 Nramp sequences for *Escherichia coli* (EcMntH), *Homo sapiens* (HsNramp1 and HsNramp2), and *Saccharomyces cerevisiae* (SMF1, SMF2, and SMF3), respectively, and without EIN2 sequence in thesespecies. The algae (*Chlamydomonas reinhardtii*) and moss (*Physcomitrella patens*) had five and four Nramps, respectively. The ancient vascular plant species *Selaginella moellendorffii* had seven Nramp homologs. However, the basal angiosperm *Amborella trichopoda* had four Nramp protein sequences. Among the eudicot species, 4–14 Nramp homolog proteins were identified. In comparison with eudicot species, monocot had just 4–10 Nramp protein sequences. A maximum number of 15 Nramps were identified in the eudicot species *Glycine max*, possibly because the copies underwent lineage-specific expansion [51].

**Figure 1:**
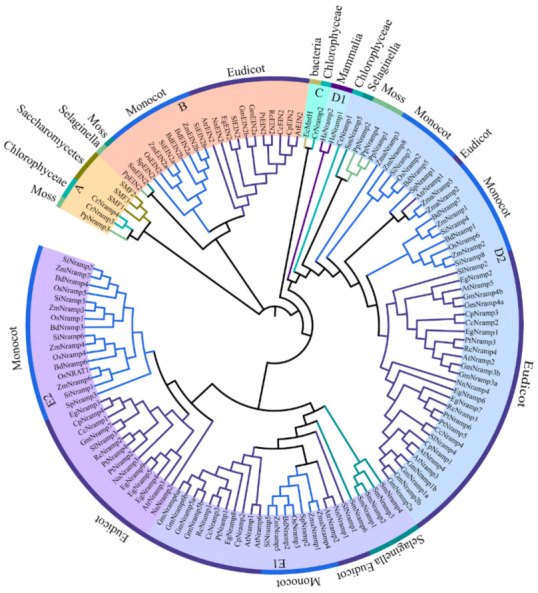
Phylogenetic analysis of 138 Nramp proteins in *S. polyrhiza* and 22 other species. The maximum likelihood tree was constructed using MEGA X (bootstrap value = 1000) based on the JTT matrix-based model. Various colored circles represent the different groups.

These *Nramp* genes encode putative proteins 412–1633 amino acid residues in length, molecular weight of 44.70–169.30 KDa, isoelectric point (pI) 4.80–9.65, 1–16 exons, and 7–13 putative transmembrane domains (TMDs) (Supplemental Materials Table S1). To estimate the phylogenetic relationships among the *Nramp* gene family, we performed phylogenetic analyses of the Nramp protein sequences based on a maximum likelihood method (Figure 1). Based on its topology, Nramps were categorized into five groups, namely A, B, C, D, and E. The moss, algae, and fungus Nramps constituted the strongly supported basal cluster A, which was similar to the major derived clusters B and C. Moreover, clusters A, B, and C were kept away from clusters D and E. Among the similar clusters, species are mainly composed of moss, algae, fungus, Selaginella, and bacteria except for the Nramps of EIN2. Cluster B and C were exclusively formed by the Nramps of EIN2, algae, and the bacteria, respectively. Sub-cluster D2 included typical Nramp homologs from all Viridiplantae species in the present study, whereas cluster E comprised Nramps exactly from vascular plants.

Four *Nramp* genes, namely *SpNramp1*, *SpNramp2*, *SpNramp3*, and *SpEIN2*, were identified in *S. polyrhiza*. Among these genes, the *SpNramp* genes carried the consensus residues GQSSTITGTYAGQF(/Y)I(/V)MQ(/G)GFLN(/D) (Table 1), a unique structural feature in a *Nramp* metal transporter family [2].

### 2.2. Gene Structure, Conserved Motif, and Chromosomal Distribution

**Figure 2:**
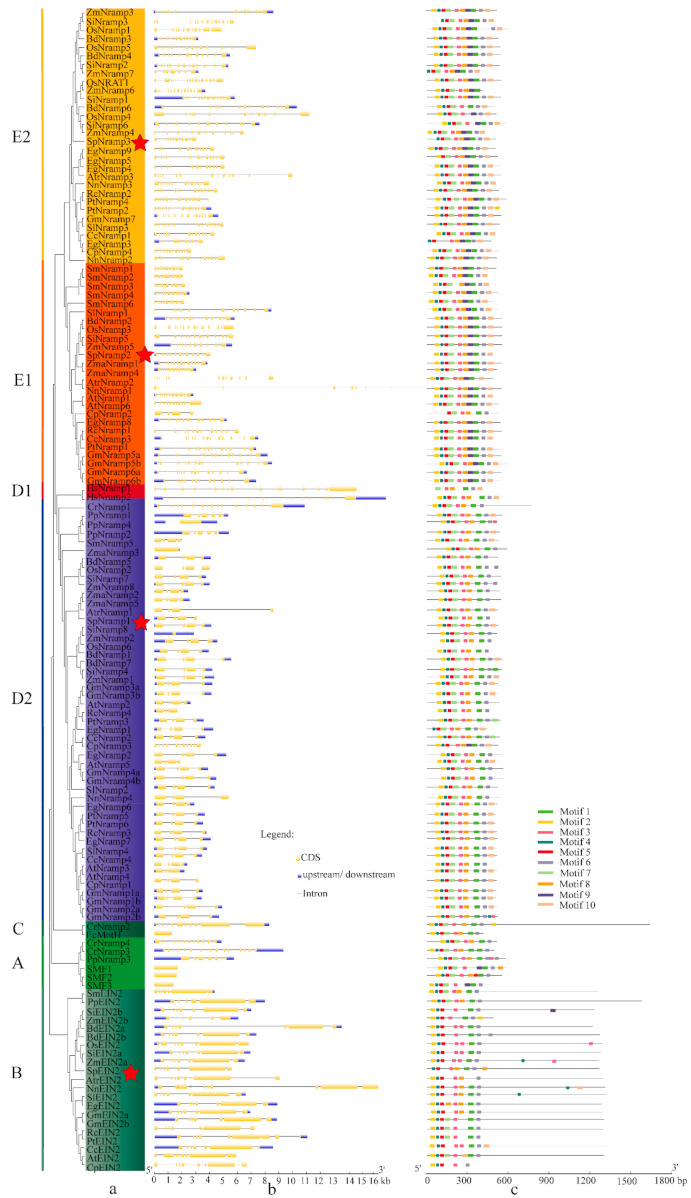
Phylogenetic relationship, gene structure, and conserved motif of *Nramp* genes. (**a**). Phylogenetic tree of 138 Nramp proteins. (**b**). Intron–exon structures of *Nramp* genes. Blue boxes represent the upstream/downstream region, and yellow boxes represent exons and black lines represent introns of Nramp proteins. (**c**). Number and distribution of conserved motifs in *Nramp* genes. Ten putative motifs were predicted by MEME, and various colors represent different colored boxes. Details of motifs are summarized in Table 1.

A gene structure map was constructed using a Gene Structure Display Server for the investigation of the gene structure feature of *Nramps* genes in all selected species (Figure 2). The evolution of multigene families was conducted on gene structural diversity to some degree [52]. According to the map, *NnNramp1* had the largest length (~ 74 kb) with 14 coding regions. *HsNramp2* had the maximum coding regions (16) with ~ 48 kb, whereas *SMF3* had the minimum length (1422 bp) coding regions (1). In addition, these genes have similar numbers of introns, exons, and gene structures on the same branch of the evolutionary tree. Interestingly, the *Nramp* genes of *EIN2*, with similar structures and motifs, were defined as EIN2 protein and belong to cluster B. To further elucidate the role of *Nramp* genes, we analyzed the conserved motifs of Nramp proteins using MEME online software, and 10 conserved motifs were identified (Figure 2). The identified conserved motifs of Nramps had a length of 23–41 amino acids. The conserved motifs revealed a closely correlation with motifs and structures. The members of the same group almost shared the same motifs and similar structures. Motif 1 had a characteristic Nramp domain in Nramp metal transporter in all the Nramp family but not in OsNramp4, HsNramp2, AtrEIN2, and CpEIN2. This was one of the reasons that we divided the EIN2 into the Nramp family. Motif 3 and 5 occurred in almost all selected species with conserved domains KTIRQAVGVVGCVIMPHNVFLHSALVQSR and LWILAEVAVIAADIPEVIGTAFALNILFH, except for SmNramp3 and HsNramp1, respectively.

**Figure 3:**
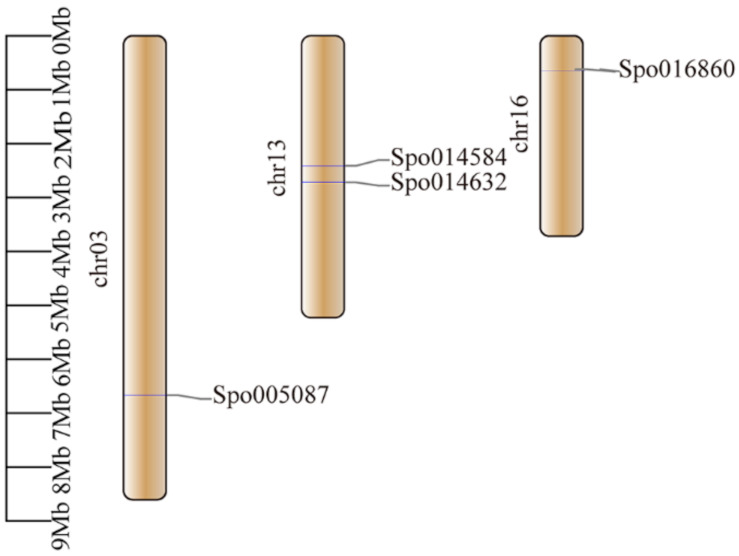
Distribution of *SpNramp* gene family on the chromosomes of *S. polyrhiza*. chromosome numbers are shown at the left of chromosome. *SpNramp* genes are labeled at the right of the chromosomes. Scale bar on the left indicates the chromosome lengths (Mb).

The members of clusters A, B, and C showed the same motif (motif 2–6) and a similar structure with a long CDS region, except for *CrNramp4*, *CrNramp3*, and *ZmEIN2b*. Sub-cluster D2 had less exon–intron number and the same structure (motif 1–8), except for *CrNramp1*, *CpNramp1*, *EgNramp1*, and *CpNramp3*. Nevertheless, sub-cluster D1 involved more exon–intron, and motif 9 was not found. In comparison with the other groups, the members of clusters A, B, C, and D were free of motif 9 with NLNPEQANCSDLDLNKASFLLKNVLGNWSSKLFAVALLAS, except for CpNramp3, SMF1, SMF3, and SiEIN2b, respectively, indicating their primitive evolutionary relationship. By contrast, motif 9 and 10 were present in cluster E, except for BdNramp6, CpNramp2, ZmNramp6, and CpNramp2, respectively, while motif 10 contained the Nramp domain IIGINVYYLSTGFVGWLIHNNLPKVANVFIGIIVFPLMALY. Besides, cluster E showed more exons, introns, and complex structures. It indicates cluster E has higher evolutionary status. The duplication and distribution of the *SpNramp* genes were analyzed in the present study with chromosome-level genome assembly of *S. polyrhiza*. Duplication events not only generate functional novelty but also lead to functional redundancy [53]. No tandem repeat and segmental duplication events were observed in *S. polyrhiza*, and this finding might be correlated with the number of *SpNramp* genes and the extent of conservation. The chromosome distribution of *SpNramp* gene was analyzed in *S. polyrhiza*. The four *SpNramp* genes were distributed across the three chromosomes, and *SpNramp1* (Spo005087) and *SpNramp3* (Spo016860) were distributed in chromosome numbers 3 and 16, respectively. *SpNramp2* (Spo014584) and *SpEIN2* (Spo014632) were distributed in chromosome number 13 (Figure 3).

**Figure 4:**
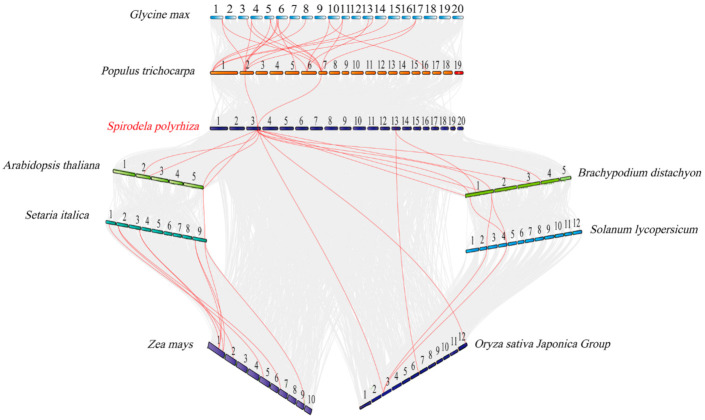
Synteny relationship of *Nramp* genes pairs among *S. polyrhiza* and eight other species. The red represents the synteny genes, and the gray lines show the collinear blocks of the plant genome. The chromosome number is labeled at the top of each chromosome.

To better know the evolutionary relationships of *Nramp* family among the nine species, we constructed a colinear map of *Nramp* family by using nine species, including Fabaceae plants (*G. max*), Salicaceae plants (*Populus trichocarpa*), Araceae plants (*S. polyrhiza*), Brassicaceae plants (*Arabidopsis thaliana*), Poaceae plants (*Setaria italica*, *Zea mays*, *Brachypodium distachyon*, *Oryza sativa*), and Solanaceae plants (*Solanum lycopersicum*) (Figure 4). Among the nine species, the colinear gene pairs were identified, as shown in Table 2.

The result implies that the continuous colinear gene pairs were found in *G. max*, *P. trichocarpa*, *S. polyrhiza*, *A. thaliana*, *S. italica*, *Z. mays* branch, *G. max*, *P. trichocarpa*, *S. polyrhiza*, *B. distachyon*, *S. lycopersicum*, and *O. sativa* branch, and we elucidate that the gene might have come from the same ancestor. Furthermore, the multiple colinear gene pairs were found in some selected species, which inferred that the genetic copies underwent lineage specific expansion.

### 2.3. Cis-Element and Promoter Analysis

**Figure 5:**
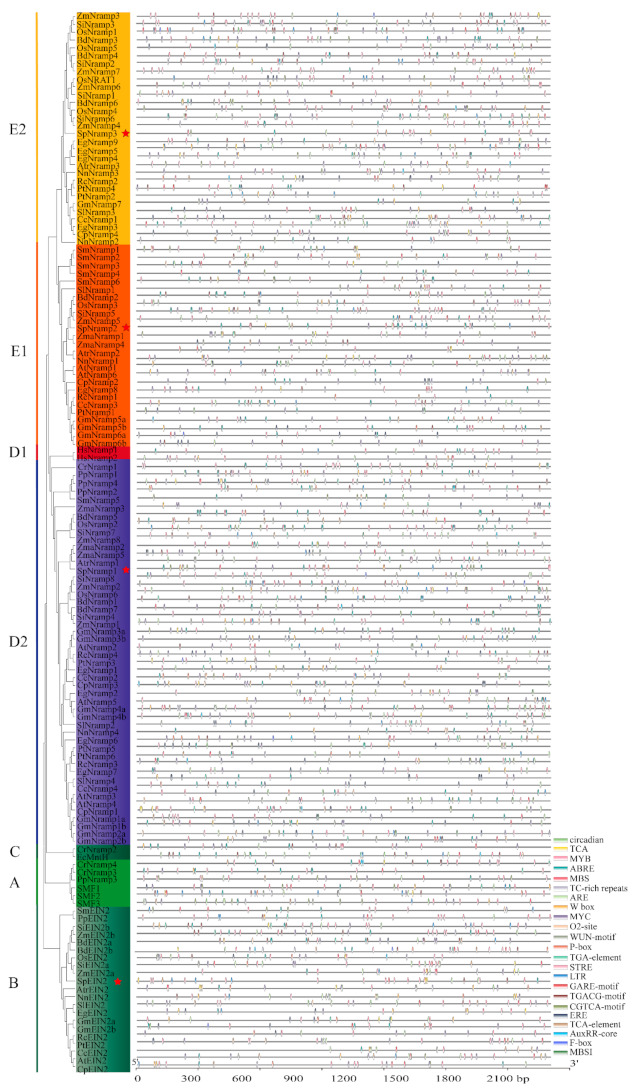
The cis-acting elements in the promoter regions in the 2000 bp upstream promoter in all selected *Nramp* genes are shown in the figure. The cis-acting elements were predicted by the online software PLANTCARE, and visualized using TBtools.

The cis-acting elements were related to gene expression in the promoter regions and play a vital role in abiotic stress. To investigate the potential regulatory and genetic expression diversification of *Nramp* genes, we extracted the upstream 2 kb promoter regions of *Nramp* genes from all selected species to analyze the cis-acting elements using the online software PLANTCARE. Afterwards, 23 cis-acting elements were selected, including the key metabolism pathway elements, hormone response elements, and stress response elements (STREs), which were visualized using TBtools (Figure 5). The MYC (CATG(T)TG) motif occurred most frequently (452 times) in all selected species involved in the stress response, while the number of F-box (CTATTCTCATT) and MBSI (aaaAaaC(G/C)GTTA) were present as the least abundant elements (both occurred 10 times). Interestingly, stress- (MYB, MYC, ARE, and MBS) and hormone- (ABRE) responsive regulatory elements were found in all species. In *S. polyrhiza*, the motif elements of circadian, TC-rich repeats, TGA-element, AuxRR-core, F-box, and MBSI were not observed. In addition, the motif element of STRE in *SpEIN2* or *SpNramp3* occurred most frequently on the *SpNramp* genes promoter regions, followed by ABRE (ACGTG) in *SpNramp2*. The stress response elements of MYB, MYC, and STRE were the most common elements in the promoter of the *SpNramp* metal transporter family, indicating that the promoter of *SpNramp* metal transporters could be engaged in stress responsiveness.

The element of ARE, a cis-acting regulatory element essential for the anaerobic induction, was distributed in all *SpNramp* metal transporters. The element of ABRE, TGACG-motif, CGTCA-motif, and TCA-element were involved in hormone-response. Among these hormone-responsive elements, the TGACG and CGTCA motifs were involved in the MeJA-responsiveness, and the ABRE and TCA-element were involved in the abscisic acid and salicylic acid responsiveness, respectively. Furthermore, GARE-motif and ERE elements were engaged in gibberellin-responsiveness, the MYB binding site of MBS was involved in drought-inducibility, and the W-box element was involved in injury-responsiveness. The O2- site and LTR elements, a cis-acting regulatory element, participated in zein metabolism regulation and low-temperature responsiveness, respectively.

**Figure 6:**
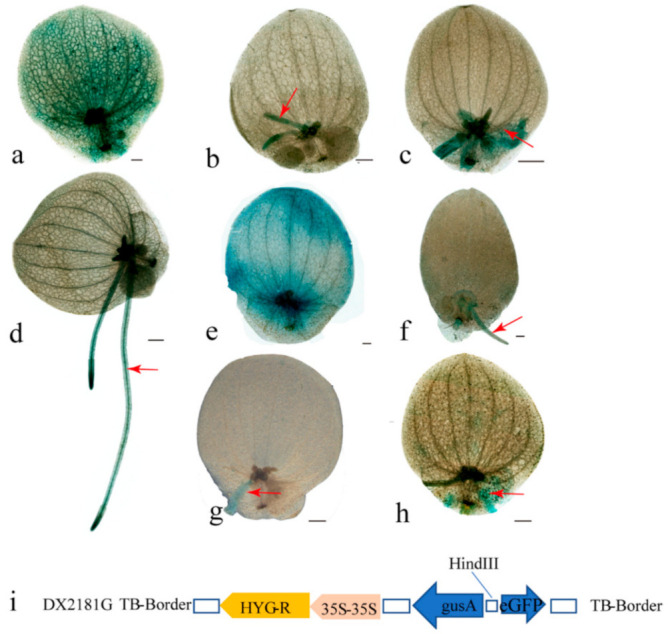
Histochemical analysis of GUS activity and the vector of frame for promoter. GUS staining in each representative transformed line constructed using promoter-GUS. GUS staining of transgenic lines *SpNramp1* (**a**–**c**), *SpNramp2* (**d**), (**e**), and *SpNramp3* (**f**–**h**) under 50 μM Cd^2+^ stress. (**a**–**c**) represent the *SpNramp1* that is mainly distributed in frond, root, and joint between mother and daughter frond. (**d**,**e**) represent the *SpNramp2* that is mainly accumulated on the root and frond. (**f**–**h**) indicate the *SpNramp3* that is focused in the root and joint between mother and daughter frond. (**i**). Vector of promoters. *SpNramp1*, *SpNramp2*, and *SpNramp3* are inserted on HindIII sites.

To investigate the tissue-specific expression pattern of *SpNramp* genes, we constructed the *SpNramps*: GUS vector and transferred them into *S. polyrhiza*. Then histochemical GUS staining was carried out as shown in Figure 6. We detected the GUS staining under the two following conditions: one group for the control condition was treated with normal 1/2 MS liquid medium, the other group was assessed under 50 μM Cd^2+^ hydroponics with 1/2 MS liquid medium conditions in *S.polyrhiza* for 7 days. The GUS activity was significantly induced in treated groups but there was almost no GUS staining in the control group. Therefore, we selected 1/2 MS liquid medium containing 50 μM Cd^2+^ for 7 days for semithin section analysis. The result showed *SpNramp1* was distributed in the root, frond, and joint between mother and daughter frond in most lines. *SpNramp2* was mainly focused on the root and frond. *SpNramp3* was widely distributed in the root and joint between mother and daughter frond.

### 2.4. Expression Profiles of Nramp Genes in Response to Different Stress in S. polyrhiza

**Figure 7:**
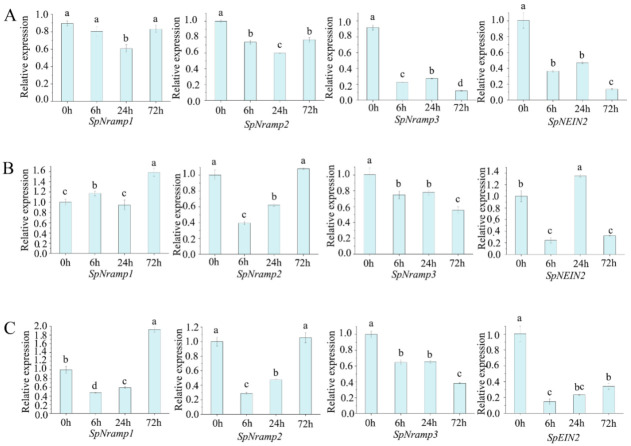
Expression patterns of four *SpNramp* genes under three abiotic stress. Quantitative RT-PCR was used to analyze the expression levels of each *SpNramp* gene. The X-axis represents the RNA samples from *S. polyrhiza* under different treatments at different time points (0, 6, 24, and 72 h). The Y-axis represents the relative expression levels of *SpNramp* genes by 2^−ΔΔCt^ method. (**A**) is treated by 50 μM Cd^2+^. (**B**,**C**) contains 50 μM Cd^2+^ under Fe^2+^ or Mn^2+^ deficiency, respectively. The *Actin* (ACT) gene was used as an internal control. The 2^−ΔΔCt^ was carried out to calculate the *SpNramp* genes’ relative expression. Different letters represent significant differences as determined by Duncan’s multiple range test (*p* ≤ 0.05). Error bars, mean ± SD.

To further determine the role of the *SpNramp* genes under stress, we used qRT-PCR to detect the gene expression. The amplified agarose gel of internal reference and target genes for qRT-PCR were shown in Supplemental Materials Figure S1. Different groups were used to examine the expression levels of *SpNramp* genes under four abiotic stress conditions. We used ANOVA at a significance threshold of *p* ≤ 0.05, expression variation among each experiment was determined with Duncan’s multiple range test, and variables marked with different letters indicate a significant difference. The results of qRT-PCR are shown in Figure 7. Under 50 μM Cd^2+^, all relative expression levels were downregulated, especially *SpNramp3* and *SpEIN2*. In comparison with the control, the expression level of *SpNramp3* and *SpEIN2* was higher than five-fold at 72 h. Moreover, the relative expression level of *SpNramp1* and *SpNramp2* initially decreased and then increased, whereas *SpNramp3* and *SpEIN2* decreased (6 h) and then increased (24 h), and finally decreased again (72 h). Under Fe^2+^ deficiency treatments, the *SpNramp* genes were differentially expressed under the same stress. *SpNramp1* was upregulated, except at 24 h, whereas *SpNramp2* initially decreased and then increased, and its relative expression level was higher than that of control. *SpNramp3* depicts a negatively regulated trend. *SpEIN2* was downregulated after treatment for 6 h, and then its expression increased significantly at 24 h. Finally, the expression level dropped dramatically at 72 h. Among the Mn^2+^ deficiency group, the initially expression level of *SpNramp1* decreased and then increased significantly. Moreover, *SpNramp2* followed a similar trend, whereas *SpNramp3* was negatively regulated. The expression of *SpEIN2* dropped dramatically and then slightly increased. Some *SpNramp* genes were upregulated under Fe^2+^ or Mn^2+^ deficiency, which suggests that they could play a vital role in response to metal cation deficiency.

## 3. Discussion

In the present study, we carried out bioinformatics analysis for all *Nramp* genes in 22 species, because gene organization might lead to functional divergence [54]. Four *Nramp* genes were identified from *S. polyrhiza,* and a phylogenetic tree was constructed with other *Nramp* genes from all selected species. Then *Nramp* genes were classified into five clusters as previously described [51]. Based on conserved motif and gene structure (exon/intron) analyses, similar motif and gene structure compositions were divided into the same cluster in the phylogenetic tree, indicating that the same subfamily had similar function (Figure 2).

The members of clusters A, B, and C were located in the phylogenetic tree root, and most of them had fewer motif and a long CDS region, and the results are similar to previous studies [55]. Additionally, similar structure features and motif arrangements were shown in D and E subfamily members. The D subfamily included fewer exon–intron and similar motif features that are greater than those of A, B, and C subfamilies. However, subfamily E had more exons, similar motif arrangements, and greater exon number than subfamily D (Figure 2). This result further confirmed the classifications of the *Nramp* genes. Previous studies had defined plant genes as high expression levels with the following features: more and longer introns, less compact, and a larger primary transcript, while it was the opposite for animals [56,57]. Jeffares et al. (2008) reported that the genes contain fewer introns among yeasts, thale cress, and mice, and this condition might be caused by rapid activated genes in response to all kinds of environmental stress [58]. Therefore, we speculate that it is on the other way in plants, and it is consistent with the result of the current study. In the present study, the phylogenetic tree showed that the intron length of *Nramp* transporter genes increased with evolution in plants, whereas the intron number of *EIN* family remained almost unchanged (4–8). Similarly, the number of motifs of the *Nramp* transporter genes also increased through evolution in general while the number of motifs of *EIN* family remained stable. Collectively, the change in the number of motifs and introns demonstrates that the plants generated a protective measure to adapt to the environment under stress.

Several previous trials showed that the expansion and evolution of gene families depend on the whole genome, segmental, tandem, and gene duplication events [59,60,61,62]. Tandem duplicate events had a higher probability with a higher level of complementarity compared with segmental duplicates [63]. Moreover, the tandem and segmental duplication events were the main duplication patterns [64]. Previous reports indicated the presence of one tandem duplicated pair and one segmental duplication pair in *T. cacao*, six pair duplicated blocks in *G. max,* one syntenic block with the paralogous pair in *O. sativa*, and two segmental duplication pairs in *A. thaliana* [51,65]. However, the findings showed that no tandem duplication, segmental duplication, and genomic collinearity events were present in *S. polyrhiza*. These findings probably resulted from the following reasons. First, *S. polyrhiza*, as a basal monocot with small genome (158 Mb), was located in a relatively primitive evolutionary position and did not contain a specific expansion [41,51]. Then, gene loss events might have occurred. Moreover, only four *SpNramp* genes were observed, thus minimizing the possibility for gene duplication events. To investigate the evolutionary relationship of *Nramp* genes, we performed genomic collinearity analysis in nine species and found many colinear gene pairs. In these colinear genes in nine species, continuous colinear gene pairs were observed, which indicated that the *Nramp* genes were highly conserved, thus representing one of the origins of homologous genes.

The cis-acting elements play a specific role in regulating gene transcription and expression in plants [66]. The promoter regions of *Nramps* contained many stress response elements (LTR, ARE, MBS, MYB, MYC, WUN-motif, TC-rich repeats, W-box, STRE, and F-box) and hormone response elements (ABRE, AuxRR-core, GARE-motif, P-box, CGTCA-motif, TGACG-motif, TCA-element, and TGA-element). Hence, *Nramp* genes may be involved in response to multiple stresses.

*OsNramp1* and *OsNramp5* were mainly expressed in leaves and roots, and *OsNramp1* was induced by Cd treatment and Fe starvation [67,68], indicating that some *Nramp*s might be inducible promoters. In the present study, the transformed lines carried out histochemical staining with various organs throughout plant development under a control or 50 μM Cd^2+^ group. Then we monitored the GUS staining in *S. polyrhiza*. GUS staining was present in many identified transformed lines without obvious distribution pattern under treatment (Figure 6). Therefore, we speculate that the promoter of *SpNramp*s may be an inducible promoter and not tissue specific.

To obtain further insight into the transcript levels, we determined the expression of *SpNramp* genes under different conditions using qRT-PCR. The experimental data indicated that all the *SpNramp* gene families were negatively regulated under 50 μM Cd^2+^ treatments. However, the expression of *SpNramp* genes were generally upregulated under some limiting ions, such as Fe^2+^ or Mn^2+^. Several similar features have been reported in the paralog genes, and the *AtNramp1* was upregulated in response to Fe^2+^ starvation [17,19]. A similar phenomenon was observed in rice, in which *OsNramp5* was upregulated under Fe^2+^ deficiency [24]. Under Fe^2+^ limited conditions, *SpEIN2* was significantly upregulated at 24 h, and then significantly downregulated. The main reason for this result was that the *EIN2* was a sense divalent cation, which monitors the physiological state in the cell or tissue and then integrates the signal with ethylene stimuli [16,30]. Beyond functioning in Fe transport, it also could be induced by Mn^2+^ starvation, and its expression can be upregulated [11]. As shown in Figure 7, *SpNramp1* was significantly upregulated, *SpNramp2* was slightly upregulated, whereas *SpNramp3* and *SpEIN2* were significantly downregulated, which proved that *SpNramp1* and *SpNramp2* could be induced by Mn deficiency. Collectively, *SpNramp1* was significantly upregulated by Mn deficiency or Fe deficiency, whereas *SpNramp3* was sharply downregulated in response to both stress conditions. In addition, all *SpNramp* genes responded to heavy metal or heavy metal under Mn or Fe deficiency, which implies that the *Nramp* genes in *S. polyrhiza* might have the function of metal ions transport. To further verify the function of the *SpNramp* genes, a series of gene overexpression and knockdown tests are essential.

## 4. Materials and Methods

### 4.1. Identification of Nramp Genes in S. polyrhiza

All non-redundancy *Nramp* sequences and genome databases of *O. sativa*, *Nelumbo nucifera cerevisiae*, *H. sapiens*, *E. coli*, *P. trichocarpa*, *Zostera marina*, *Carica papaya*, *Citrus clementina*, *Ricinus communis*, *Eucalyptus grandis*, *P. patens*, *Z. mays*, *S. polyrhiza*, *S. moellendorffii*, *S. italica*, *S. lycopersicum*, *G. max*, *C. reinhardtii*, *B. distachyon*, and *A. thaliana* were downloaded from the NCBI (https://www.ncbi.nlm.nih.gov/, accessed on 28 February 2021) and Phytozome (https://phytozome.jgi. doe.gov/pz/, accessed on 28 February 2021). The Hidden Markov Model (HMM) profile of the Nramp domain (PF01566) was extracted from the Pfam database (http://pfam.xfam.org/, accessed on 28 February 2021). The Nramp proteins of *A. thaliana* and *O. sativa* were used as query sequences in BLASTP to identify the orthologs in these species. To identify the Nramps conserved protein domains, we used the HMMER program (http://hmmer.org/, accessed on 28 February 2021) [69]. The protein domains of all sequences were screened and confirmed using the SMART (http://smart.embl.de/smart/batch.pl, accessed on 28 February 2021), CDD (https://www.ncbi.nlm.nih.gov/cdd/, accessed on 28 February 2021), and Pfam databases.

### 4.2. Phylogenetic, Gene Feature, Gene Duplication, Cis-Acting Element, Classification, and Conserved Motif Analysis

Multiple alignments of Nramp domains were performed using ClustalW [70], and displayed by TBtools software [71]. A maximum likelihood phylogenetic tree was constructed by MEGA X (v10.2.2) [72] with a minimum bootstrap of 1000 replicates, visualized and annotated using iTOL [73]. The *Nramp* genes were classified into different groups based on their topology. The PI and MW of the Nramps sequences were computed by the online ExPASy-ProtParam tool (http://web.expasy.org/protparam/, accessed on 3 March 2021) [74]. TMDs were predicted by the online software TMHMM Server v.2.0 (http://www.cbs.dtu.dk/services/TMHMM/, accessed on 3 March 2021) [75]. The subcellular localization of each Nramps protein was predicted using the online CELLO v2.5 server (http://cello.life.nctu.edu.tw/, accessed on 3 March 2021), pLoc-mPlant (http://www.jci-bioinfo.cn/pLoc-mPlant/, accessed on 3 March 2021) and PSORT (https://psort.hgc.jp/, accessed on 3 March 2021) [76,77,78]. Exon–intron structure information for *Nramp* genes were constructed using the online tools Gene Structure Display Server 2.0 (http://gsds.cbi.pku.edu.cn/, accessed on 3 March 2021) [79]. The MEME program (http://meme-suite.org/, accessed on 3 March 2021) was employed to identify the conserved motifs in Nramp proteins with the maximum number of motifs set as 10 [80]. The cis-acting regulatory DNA elements (cis-elements) in the promoter regions (2000 bp upstream of the start codon) of *Nramp* genes were predicted and analyzed using PLANTCARE (http://bioinformatics.psb.ugent.be/webtools/plantcare/html/, (accessed on 3 March 2021) and visualized using the TBtools [71,81]. The chromosomal distribution of the *Nramp* genes on *S. polyrhiza* was determined from the genome annotation gff3 file. Synteny, collinearity, and segmental duplication pairs were analyzed by MCScanX and MCScanX-transposed [82,83]. We also identified the syntenic relationships among the nine species using MCScanX and displayed by TBtools.

### 4.3. Promoter Analysis

A series of *SpNramp1*, *SpNramp2*, and *SpNramp3* promoter fragments (956, 1144, and 2141 bp) were amplified by PCR with the template of *S. polyrhiza* genome. All *SpNramp* genes of primers are generated by primer–primer 5 (Supplemental Materials Table S2). All amplified sequences were inserted into the HindIII sites of DX2181G (Figure 6i) by homologous recombination (TsingKe, Wuhan, China). The recombinant plasmids of *SpNramp* genes were introduced into *Agrobacterium tumefaciens* strain LBA4404 using the liquid nitrogen freeze–thaw method [66]. Consequently, agrobacterium harboring the plasmids (OD_600_ = 0.5–1) were transferred into *S. polyrhiza* by the frond transformation system. After obtaining independent transgenic lines, 20 transgenic lines were treated with 50 μM Cd^2+^ and then applied for GUS staining analysis. The histological staining of GUS was assayed using the method of Jefferson et al. (1987) with slight modifications [84]. Fronds were placed directly in a staining solution containing 0.96 mM 5-bromo-4-chloro-3-indolyl-β-D-glucuronic acid sodium salt (X-Gluc, Sigma, St. Louis, MO, USA), 0.5 mM K_3_[Fe (CN)_6_], 0.5 mM K_4_[Fe (CN)_6_], 5 mM ethylene diamine tetraacetic acid (EDTA), and 100 mM KPO_4_ buffer (pH 7.0) for 40–60 min, vacuum-infiltrated, and incubated for 16–24 h in the dark at 37 °C. Then, the chlorophyll was removed from fronds using 75% ethanol, and the samples were photographed using Leica Z16 microscope (Germany).

### 4.4. Expression Profile Analysis of SpNramp Genes

*S. polyrhiza* strain 5543 was collected from East Lake (30°32′ N, 114°21′ E) at the city of Wuhan, Hubei Province, China. The plants were cultured in 1/2 MS medium under the conditions described in Murashige and Skoog. (1962) with 16 h/8 h photoperiod (day/night) and temperature of 25 °C/15 °C (day/night) [85]. To display the expression levels of *SpNramp* genes under different abiotic stresses, we have set the four following groups: (1) control, (2) Fe^2+^ starvation and 50 μM Cd^2+^, (3) Mn^2+^ starvation and 50 μM Cd^2+^, and (4) 50 μM Cd^2+^ under the same conditions described above. Each group included three biological replicates. All plants were sampled at each time point (0, 6, 24, and 72 h) and then immediately frozen in liquid nitrogen and stored at −80 °C for further analysis.

The oligonucleotide primers of *SpNramp* genes were designed by primer–primer 5 (Supplemental Materials Table S2). The Ominiplant RNA kit (CoWin Biosciences, Beijing, China) was used to extract the total RNA. The first-strand cDNA was synthesized using the PrimeScript^TM^ RT reagent kit (TaKaRa, Dalian, China). Bio-Rad CFX96 touch real time PCR system (Bio-Rad, Hercules, CA, USA) was used to run qRT-PCR with the TB Green^®^ Premix Ex Taq^TM^ kit (TaKaRa, Dalian, China). The qPCR program under the following conditions: 10 min at 95 °C, 40 cycles of 95 °C for 15 s, 59 °C for 30 s, and 72 °C for 30 s. A melting process at 60–95 °C was designed to generate the melting curve. The *Actin* (ACT) gene was used as an internal control. The 2^−ΔΔCt^ method was employed to calculate the *SpNramp* genes’ relative expression [86]. Data were presented as the means ± standard deviations. Analysis of variance was carried out by SPSS 24.0 software using one-way analysis of variance (ANOVA).

## 5. Conclusions

Notably, the study was the first to elaborate in detail about the *Nramp* gene family information in genome-wide in *S. polyrhiza*. A series of promoter analysis indicated that there exists many stress and hormone response elements, which play a vital role in metal stress responses. GUS staining results indicate that *SpNramp1*, *SpNramp2*, and *SpNramp3* were highly expressed in the root, indicating that the roots are involved in ions uptake. Furthermore, expression patterns demonstrate that *SpNramp1*, *SpNramp2,* and *SpNramp3* were significantly induced by Fe or Mn starvation, but suppressed by single Cd treatment. Collectively, the study provided a foundation for the mechanisms research of heavy metal element absorption and transport in *S. polyrhiza*.

## Figures and Tables

**Table 1:** List of the putative motifs of Nramp proteins.

Motif	Logo	Best Possible Match	*E*-Value	Width
Motif 1	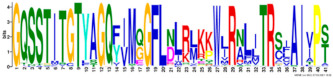	GQSSTITGTYAG QFIMGGFLNLRL KKWMRALITRS CAIVPT	1.6e-3723	41
Motif 2	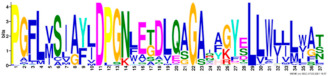	PGFLMSIAFLDP GNLEGDLQAGA IAGYSLLWLLM WAT	3.6e-3053	37
Motif 3	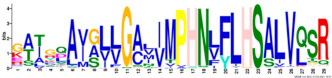	KTIRQAVGVVG CVIMPHNVFLH SALVQSR	9.2e-2530	29
Motif 4	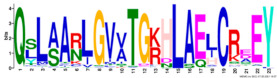	QLLSARLGVVT GRHLAEHCREE Y	2.6e-2207	23
Motif 5	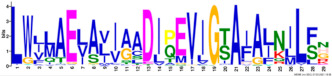	LWILAEVAVIAA DIPEVIGTAFAL NILFH	3.4e-2250	29
Motif 6	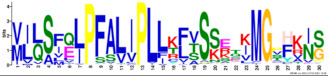	MILSFELPFALIP LLKFSSSRTKM GPHKNS	1.4e-2224	30
Motif 7	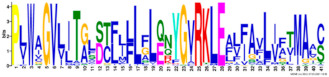	PLWAGVIITALD CFIFLFLENYGV RKLEAVFAVLIA TMALS	1.7e-2825	41
Motif 8	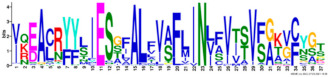	VQEALRYYSIES TIALVVSFMINL FVTTVFAKGFY GT	1.5e-2317	37
Motif 9	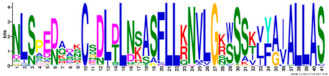	NLNPEDQANCS DLDLNKASFLL KNVLGNWSSK LFAVALLAS	8.9e-1336	41
Motif 10	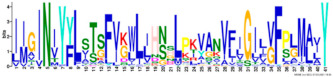	IIGINVYYLSTG FVGWLIHNNLP KVANVFIGIIVF PLMALY	4.3e-1126	41

**Table 2:** The colinear gene pairs.

Species 1	Species 2	Colinear Gene Pairs
*Glycine max*	*Populus trichocarpa*	*GmNramp1a*: *PtNramp3*, *GmNramp1a*: *PtNramp5*, *GmNramp1b*: *PtNramp3*, *GmNramp1b*: *PtNramp5*, *GmNramp2a*: *PtNramp3*, *GmNramp2a*: *PtNramp5*, *GmNramp2b*: *PtNramp3*, *GmNramp2b*: *PtNramp5*, *GmNramp3a*: *PtNramp3*, *GmNramp3a*: *PtNramp5*, *GmNramp3b*: *PtNramp3*, *GmNramp3b*: *PtNramp5*, *GmNramp6a*: *PtNramp1*, *GmNramp6b*: *PtNramp1*
*Populus trichocarpa*	*Spirodela polyrhiza*	*PtNramp3*: *SpNramp1*, *PtNramp5*: *SpNramp1*
*Spirodela polyrhiza*	*Arabidopsis thaliana*	*SpNramp1*: *AtNramp3*, *SpNramp1*: *AtNramp4*
*Arabidopsis thaliana*	*Setaria italica*	*AtNramp4*: *SiNramp8*
*Setaria italica*	*Zea mays*	*SiNramp2*: *ZmNramp3*, *SiNramp2*: *ZmNramp7*, *SiNramp2*: *ZmNramp8*, *SiNramp4*: *ZmNramp1*, *SiNramp7*: *ZmNramp1*, *SiNramp8*: *ZmNramp1*, *SiNramp8*: *ZmNramp8*
*Spirodela polyrhiza*	*Brachypodium distachyon*	*SpNramp1*: *BdNramp1*, *SpNramp1*: *BdNramp5*, *SpNramp1*: *BdNramp7*, *SpNramp2*: *BdNramp2*
*Brachypodium distachyon*	*Solanum lycopersicum*	*BdNramp5*: *SlNramp4*, *BdNramp1*: *SlNramp2*, *BdNramp5*: *SlNramp2*
*Solanum lycopersicum*	*Oryza sativa*	*SlNramp2*: *OsNramp2*, *SlNramp4*: *OsNramp2*
*Spirodela polyrhiza*	*Oryza sativa*	*SpNramp1*: *OsNramp2*, *SpNramp1*: *OsNramp6*, *SpNramp2*: *OsNramp3*, *SpEIN2*: *OsEIN2*

## Data Availability

The datasets used and/or analyzed during the current study are available from the corresponding author on reasonable request.

## Supplementary Materials

The following are available online at https://www.mdpi.com/article/10.3390/ijms22126414/s1.
