# Supplementary material for: Genome-Wide Identification of the Nramp Gene Family in Spirodela polyrhiza and Expression Analysis under Cadmium Stress
Source: Int J Mol Sci. 2021 Jun 15;22(12):6414. doi: 10.3390/ijms22126414 (PMC8232720; doi:10.3390/ijms22126414)
Supplement: Supplementary file 1 [file ijms-22-06414-s001.zip › Figure S1. The amplified agarose gel of internal reference and target genes for qRT-PCR..pdf]

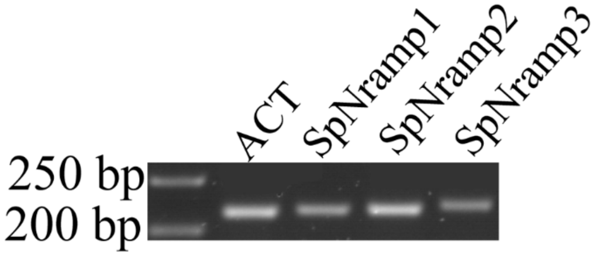

Figure S1. The amplified agarose gel of internal reference and target genes for qRT-PCR. From left to right, they are marker, ACT (135 bp), SpNramp1 (146 bp), SpNramp2 (148 bp), and SpNramp3 (156 bp).
