# Supplementary material for: Genome-Wide Identification of the Nramp Gene Family in Spirodela polyrhiza and Expression Analysis under Cadmium Stress
Source: Int J Mol Sci. 2021 Jun 15;22(12):6414. doi: 10.3390/ijms22126414 (PMC8232720; doi:10.3390/ijms22126414)
Supplement: Supplementary file 1 [file ijms-22-06414-s001.zip › Table S1. Feature of Nramp genes in all selected species.pdf]

Table S1 Feature of *Nramp* genes in all selected species

Designation	Gene ID	Length(aa)	TMHs	MW(KD)	pI	Exon	Species
AtrNramp1	AmTr_v1.0_scaffold00001.588	521	11	58.58101	5.75	4	Amborella trichopoda
AtrNramp2	AmTr_v1.0_scaffold00092.96	482	10	53.29511	9.1	12	Amborella trichopoda
AtrNramp3	AmTr_v1.0_scaffold00203.5	544	12	60.21296	8.47	13	Amborella trichopoda
AtrEIN2	AmTr_v1.0_scaffold00148.35	1291	13	142.79022	5.88	7	Amborella trichopoda
AtNramp1	NM_001335016.1	532	12	58.30396	8.94	11	Arabidopsis thaliana
AtNramp2	NM_103618.3	530	9	59.15919	5.13	4	Arabidopsis thaliana
AtNramp3	NM_127879.4	509	11	56.8797	5.18	4	Arabidopsis thaliana
AtNramp4	NM_126133.4	512	12	57.12703	5.05	3	Arabidopsis thaliana
AtNramp5	NM_117995.2	530	10	59.52328	4.89	4	Arabidopsis thaliana
AtNramp6	NM_101464.4	527	12	57.98717	8.33	13	Arabidopsis thaliana
AtEIN2	NM_120406.5	1294	11	141.48505	5.67	8	Arabidopsis thaliana
BdNramp1	XM_003559636.4	526	10	58.51689	5.38	4	Brachypodium distachyon
BdNramp2	XM_024457271.1	551	12	60.56848	8.46	13	Brachypodium distachyon
BdNramp3	XM_003561220.4	523	11	57.39498	8.62	13	Brachypodium distachyon
BdNramp4	XM_003557277.4	544	11	59.60245	6.73	13	Brachypodium distachyon
BdNramp5	XM_003558487.4	518	11	57.29746	5.69	4	Brachypodium distachyon
BdNramp6	XR_002963009.1	489	10	54.04109	8.66	13	Brachypodium distachyon
BdNramp7	XM_003579195.3	551	11	60.23059	5.05	4	Brachypodium distachyon
BdEIN2a	XM_024456578.1	1213	10	133.52595	6.66	7	Brachypodium distachyon
BdEIN2b	XM_003575523.4	1264	11	138.66396	6.05	7	Brachypodium distachyon
CcNramp1	Ciclev10000736m	559	12	61.35633	8.82	13	Citrus clementina

CcNramp2	Ciclev10000826m	532	11	59.07307	5.26	4	Citrus clementina
CcNramp3	Ciclev10025318m	543	12	59.51103	8.69	13	Citrus clementina
CcNramp4	Ciclev10025394m	512	10	57.00303	5.38	4	Citrus clementina
CcEIN2	Ciclev10010923m	1317	12	145.99773	5.35	8	Citrus clementina
CpNramp1	PAC:16423664	509	11	56.26922	5.42	4	Carica papaya
CpNramp2	PAC:16413399	527	9	57.54379	5.36	5	Carica papaya
CpNramp3	PAC:16424200	522	10	58.02517	8.52	13	Carica papaya
CpNramp4	PAC:16424201	526	12	57.86301	6.63	13	Carica papaya
CpEIN2	PAC:16419119	1109	7	120.75937	5.75	8	Carica papaya
CrNramp1	Cre05.g248300.t1.1	767	11	79.39657	5.26	15	Chlamydomonas reinhardtii
CrNramp2	Cre07.g315200.t1.1	1633	10	162.65027	9.21	11	Chlamydomonas reinhardtii
CrNramp3	Cre07.g321951.t1.1	496	9	53.33468	9.25	15	Chlamydomonas reinhardtii
CrNramp4	Cre17.g707700.t1.2	513	11	55.54077	6.73	13	Chlamydomonas reinhardtii
EcMntH	gene-b2392	412	11	45.37309	9.65	1	Escherichia coli
EgNramp1	Eucgr.F00133.1	441	10	49.10735	8.96	1	Eucalyptus grandis
EgNramp2	Eucgr.F02273.1	551	10	61.22868	7.32	4	Eucalyptus grandis
EgNramp3	Eucgr.F03813.1	470	7	51.95079	8.8	11	Eucalyptus grandis
EgNramp4	Eucgr.F04336.1	542	12	59.03365	8.62	13	Eucalyptus grandis
EgNramp5	Eucgr.F04337.1	519	10	56.49213	6.8	12	Eucalyptus grandis
EgNramp6	Eucgr.H02266.1	519	10	56.59657	4.92	4	Eucalyptus grandis
EgNramp7	Eucgr.I00738.1	520	12	57.43132	5.63	4	Eucalyptus grandis
EgNramp8	Eucgr.J03115.1	537	12	58.85753	8.96	13	Eucalyptus grandis
EgNramp9	Eucgr.L02462.1	500	12	54.61454	8.39	12	Eucalyptus grandis
EgEIN2	Eucgr.K01247.1	1277	11	140.15125	5.81	7	Eucalyptus grandis

GmNramp1a	NM_001357773.1	507	11	56.4724	5.45	4	Glycine max
GmNramp1b	NM_001357777.1	506	11	56.31462	5.15	4	Glycine max
GmNramp2a	NM_001357778.1	516	10	57.41329	5.32	4	Glycine max
GmNramp2b	NM_001249798.2	516	11	57.46784	5.04	4	Glycine max
GmNramp3a	NM_001357779.1	518	11	57.56445	5.3	4	Glycine max
GmNramp3b	XM_003527681.4	522	11	58.13405	5.19	4	Glycine max
GmNramp4a	XM_003529854.4	557	11	62.59558	4.9	4	Glycine max
GmNramp4b	XM_014768591.2	559	11	62.74865	4.8	4	Glycine max
GmNramp5a	XM_003530227.3	545	12	60.00772	8.65	13	Glycine max
GmNramp5b	XM_006585546.3	588	12	65.26629	9.11	14	Glycine max
GmNramp6a	XM_003543653.4	544	12	59.90867	8.61	13	Glycine max
GmNramp6b	XM_003546866.4	546	12	60.08052	8.84	13	Glycine max
GmNramp7	XM_003526597.3	544	12	59.4101	8.96	13	Glycine max
GmEIN2a	XM_006576961.3	1287	9	141.05002	5.78	7	Glycine max
GmEIN2b	XM_006588735.2	1298	11	143.0908	5.67	7	Glycine max
NnNramp1	XM_010260352.2	545	12	59.90638	8.59	14	Nelumbo nucifera
NnNramp2	XM_010267694.1	511	10	55.94588	9.11	12	Nelumbo nucifera
NnNramp3	XM_010271952.2	563	12	62.70839	7.33	13	Nelumbo nucifera
NnNramp4	XM_010273260.2	534	11	59.45671	5.85	4	Nelumbo nucifera
NnEIN2	XM_010255609.2	1306	11	143.27897	5.37	8	Nelumbo nucifera
OsNramp1	LOC_Os07g15460.1	600	10	66.3476	9.01	12	Oryza sativa Japonica Group
OsNramp2	LOC_Os03g11010.1	524	10	57.8218	6.2	4	Oryza sativa Japonica Group
OsNramp3	LOC_Os06g46310.2	550	12	60.60216	8.52	14	Oryza sativa Japonica Group

OsNramp4	LOC_Os01g31870.1	508	9	55.43684	7.96	12	Oryza sativa Japonica Group
OsNramp5	LOC_Os07g15370.1	538	12	59.39197	6.9	13	Oryza sativa Japonica Group
OsNramp6	LOC_Os12g39180.1	535	9	59.22809	5.48	4	Oryza sativa Japonica Group
OsNRAT1	LOC_Os02g03900.1	545	12	60.0114	7.37	14	Oryza sativa Japonica Group
OsEIN2	LOC_Os07g06130.2	1281	10	138.89434	5.83	8	Oryza sativa Japonica Group
PpNramp1	PAC:32970512	551	12	61.29764	5.47	6	Physcomitrella patens
PpNramp2	PAC:32932855	534	12	59.84696	5.36	5	Physcomitrella patens
PpNramp3	PAC:32924238	579	12	65.0711	5.46	7	Physcomitrella patens
PpNramp4	PAC:32932314	513	11	56.04957	9.14	2	Physcomitrella patens
PpEIN2	PAC:32951864	1575	11	169.30432	5.4	6	Physcomitrella patens
PtNramp1	XM_006368452.2	541	12	59.42295	8.75	13	Populus trichocarpa
PtNramp2	XM_006386304.2	546	12	59.9214	8.66	13	Populus trichocarpa
PtNramp3	XM_002302388.3	536	11	59.34433	5.37	4	Populus trichocarpa
PtNramp4	XM_024601420.1	581	12	64.24256	8.13	14	Populus trichocarpa
PtNramp5	XM_006380609.2	505	11	56.23212	5.01	4	Populus trichocarpa
PtNramp6	XM_024605007.1	500	11	55.39811	4.95	4	Populus trichocarpa
PtEIN2	XM_024604330.1	1310	11	142.84779	5.65	9	Populus trichocarpa
RcNramp1	29588.m000852	546	12	59.93465	8.81	13	Ricinus communis
RcNramp2	29648.m002004	528	12	57.98466	8.79	13	Ricinus communis
RcNramp3	29970.m001011	509	11	56.84692	5.4	4	Ricinus communis
RcNramp4	30170.m013772	462	9	51.48123	5.54	3	Ricinus communis
RcEIN2	30078.m002320	1290	11	141.38223	5.42	7	Ricinus communis

SiNramp1	XM_004951945.2	543	10	59.91941	8.73	13	Setaria italica
SiNramp2	XM_004955849.3	532	11	58.89552	7.6	13	Setaria italica
SiNramp3	XM_004959079.3	516	11	56.57095	8.25	13	Setaria italica
SiNramp4	XM_004962946.2	546	11	59.94994	5.11	4	Setaria italica
SiNramp5	XM_022825703.1	551	12	60.24255	7.57	15	Setaria italica
SiNramp6	XM_004975255.2	581	11	63.17824	8.47	13	Setaria italica
SiNramp7	XM_004982389.3	542	7	59.91655	5.53	4	Setaria italica
SiNramp8	XM_004985206.3	522	9	57.58448	6.46	4	Setaria italica
SiEIN2a	XM_004955428.2	1272	11	138.6728	5.79	7	Setaria italica
SiEIN2b	XM_004982021.3	1228	10	134.47954	5.98	7	Setaria italica
HsNramp1	NM_000578.4	441	9	48.74417	8.2	15	Homo sapiens
HsNramp2	XM_017019356.2	561	9	61.83031	6.02	16	Homo sapiens
SlNramp1	XM_004235892.4	522	12	57.98043	8.89	13	Solanum lycopersicum
SlNramp2	NM_001321237.1	519	9	58.42	5.31	4	Solanum lycopersicum
SlNramp3	NM_001247389.1	530	12	59.05761	7.59	13	Solanum lycopersicum
SlNramp4	NM_001246841.1	509	11	57.42154	5.73	4	Solanum lycopersicum
SlEIN2	XM_010327408.3	1316	11	143.73486	5.98	7	Solanum lycopersicum
SMF1	NM_001183376.1	575	11	63.62923	6.15	1	Saccharomyces cerevisiae
SMF2	NM_001179180.1	549	11	60.1337	5.66	1	Saccharomyces cerevisiae
SMF3	NM_001181921.2	473	10	52.14087	8.39	1	Saccharomyces cerevisiae
SmNramp1	PACid:15419327	507	12	56.0968	8.51	10	Selaginella moellendorffii
SmNramp2	PACid:15422119	494	11	54.83209	9.1	10	Selaginella moellendorffii
SmNramp3	PACid:15420600	516	11	57.12462	6.4	12	Selaginella moellendorffii
SmNramp4	PACid:15414174	522	12	57.73049	9.31	13	Selaginella moellendorffii
SmNramp5	PACid:15401550	528	10	58.37842	6.51	5	Selaginella moellendorffii
SmNramp6	PACid:15419877	484	11	53.58924	8.71	12	Selaginella moellendorffii
SmEIN2	PACid:15422392	1251	12	135.45997	5.21	7	Selaginella moellendorffii

SpNramp1	Spo005087	538	11	59.66955	5.3	4	Spirodela polyrhiza
SpNramp2	Spo014584	541	12	58.90639	9.19	13	Spirodela polyrhiza
SpNramp3	Spo016860	505	11	54.75565	8.62	10	Spirodela polyrhiza
SpEIN2	Spo014632	1260	10	137.04552	5.37	7	Spirodela polyrhiza
ZmNramp1	NM_001175746.1	544	11	59.70408	4.96	4	Zea mays
ZmNramp2	XM_008682933.3	514	7	57.13786	5.77	1	Zea mays
ZmNramp3	XM_008671862.4	536	10	58.54722	8.84	13	Zea mays
ZmNramp4	XM_008672540.4	469	9	51.88155	8.94	11	Zea mays
ZmNramp5	NM_001138648.1	550	12	60.35149	8.46	13	Zea mays
ZmNramp6	NM_001347090.1	413	9	44.702	8.78	13	Zea mays
ZmNramp7	NM_001367937.1	449	10	49.40514	8.74	7	Zea mays
ZmNramp8	NM_001156808.2	516	9	57.03861	6.52	4	Zea mays
ZmEIN2a	XM_035966126.1	1258	11	137.27153	5.9	7	Zea mays
ZmEIN2b	NM_001359500.1	488	9	53.7995	6.67	5	Zea mays
ZmaNramp1	Zosma39g00370.1	545	12	60.50273	7.66	13	Zostera marina
ZmaNramp2	Zosma52g00820.1	532	10	60.1409	5.26	4	Zostera marina
ZmaNramp3	Zosma57g00220.1	587	11	66.27809	6.25	1	Zostera marina
ZmaNramp4	Zosma153g00180.1	510	12	56.4493	8.49	13	Zostera marina
ZmaNramp5	Zosma303g00140.1	543	11	60.682	5.66	4	Zostera marina
