# Supplementary material for: Genome-Wide Identification of the Nramp Gene Family in Spirodela polyrhiza and Expression Analysis under Cadmium Stress
Source: Int J Mol Sci. 2021 Jun 15;22(12):6414. doi: 10.3390/ijms22126414 (PMC8232720; doi:10.3390/ijms22126414)
Supplement: Supplementary file 1 [file ijms-22-06414-s001.zip › Table S2. The primer sequences in the study.pdf]

Table S2 The primer sequences in the study

Primer name	Primer sequence	Amplicon length	Note
pSpNramp1-F	GATCTACAGCGCTAAGCTTCGGCTCGAGCTCGCC	956 bp	The primers for promoter vector
pSpNramp1-R	CGACCTGCAGCCAAGCTTCTCCTCCTTTATATAGCAGG		
pSpNramp2-F	GATCTACAGCGCTAAGCTTGGCGGAAAACGGCGAC	1144 bp	
pSpNramp2-R	CGACCTGCAGCCAAGCTTTGCCGCCATGCCCCCT		
pSpNramp3-F	GATCTACAGCGCTAAGCTTGCTGTACTGGGGCAATGC	2141 bp	
pSpNramp3-R	TCGACCTGCAGCCAAGCTTGAGGACGCCGACCCAGAG		
ACT-F	GCCCCCAGCAGCATGA	135 bp	Located in exonic regions
ACT-R	CAAGTCCGAGTACGACGAGTCC		
SpNramp1-RT-F	GAGAAGGCCTACGAGCAGAATG	146 bp	Located in exonic regions
SpNramp1- RT-R	GCAACGCTCATCAGGAATCC		
SpNramp2- RT-F	GCTGGAGCAGGCAAATTGAT	148 bp	Located in exonic regions
SpNramp2- RT-R	CCCACGTCACCACTGATATCG		spanned intronic regions
SpNramp3- RT-F	TCTGGTTATGCCCCACAATCTC	156 bp	spanned intronic regions
SpNramp3- RT-R	ACGACGGCGATGTTGATCA		Located in exonic regions
